# (*E*)-1-(4-Amino­phen­yl)ethanone oxime

**DOI:** 10.1107/S1600536808034120

**Published:** 2008-10-22

**Authors:** Muhammad Rafiq, Muhammad Hanif, Ghulam Qadeer, Sauli Vuoti, Juho Autio

**Affiliations:** a Department of Chemistry, BZU, Multan, Pakistan; b Department of Chemistry, Quaid-i-Azam Univeristy, Islamabad 45320, Pakistan; c Department of Chemistry, University of Oulu, PO Box 3000, 90014 Finland

## Abstract

In the mol­ecule of the title compound, C_8_H_10_N_2_O, the oxime group is oriented at a dihedral angle of 5.58 (3)° with respect to the benzene ring. In the crystal structure, inter­molecular O—H⋯N and N—H⋯O hydrogen bonds link the mol­ecules, forming a three-dimensional network.

## Related literature

For general background, see: Bertolasi *et al.* (1982 ▶); Degorre *et al.* (1998 ▶). For bond-length data, see: Allen *et al.* (1987 ▶).
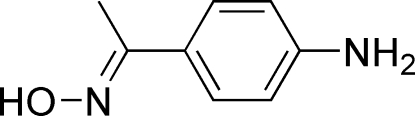


## Experimental

### 

#### Crystal data

C_8_H_10_N_2_O
*M* *_r_* = 150.18
Monoclinic, 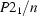

*a* = 4.8641 (2) Å
*b* = 9.2016 (3) Å
*c* = 17.1447 (7) Å
β = 95.535 (2)°
*V* = 763.78 (5) Å^3^
*Z* = 4
Mo *K*α radiation
μ = 0.09 mm^−1^
*T* = 100 (2) K
0.34 × 0.28 × 0.26 mm

#### Data collection

Enraf–Nonius KappaCCD diffractometer
Absorption correction: multi-scan (*DENZO*; Otwinowski & Minor, 1997 ▶) *T*_min_ = 0.972, *T*_max_ = 0.979
6132 measured reflections
1761 independent reflections
1483 reflections with *I* > 2σ(*I*)
*R*_int_ = 0.026

#### Refinement

*R*[*F* ^2^ > 2σ(*F* ^2^)] = 0.040
*wR*(*F* ^2^) = 0.107
*S* = 1.04
1761 reflections
113 parameters
H atoms treated by a mixture of independent and constrained refinement
Δρ_max_ = 0.24 e Å^−3^
Δρ_min_ = −0.28 e Å^−3^

### 

Data collection: *COLLECT* (Hooft, 1998 ▶); cell refinement: *DENZO* (Otwinowski & Minor, 1997 ▶) and *COLLECT*; data reduction: *DENZO* and *COLLECT*; program(s) used to solve structure: *SIR2004* (Burla *et al.*, 2005 ▶); program(s) used to refine structure: *SHELXL97* (Sheldrick, 2008 ▶); molecular graphics: *DIAMOND* (Brandenburg, 2007 ▶) and *PLATON* (Spek, 2003 ▶); software used to prepare material for publication: *SHELXL97*.

## Supplementary Material

Crystal structure: contains datablocks I, global. DOI: 10.1107/S1600536808034120/hk2552sup1.cif

Structure factors: contains datablocks I. DOI: 10.1107/S1600536808034120/hk2552Isup2.hkl

Additional supplementary materials:  crystallographic information; 3D view; checkCIF report

## Figures and Tables

**Table 1:** Hydrogen-bond geometry (Å, °)

*D*—H⋯*A*	*D*—H	H⋯*A*	*D*⋯*A*	*D*—H⋯*A*
O1—H1*O*⋯N2^i^	0.92 (2)	1.88 (2)	2.7919 (14)	169.8 (18)
N2—H2*N*⋯O1^ii^	0.916 (18)	2.165 (18)	3.0790 (13)	175.7 (15)
N2—H2*M*⋯N1^iii^	0.929 (19)	2.525 (19)	3.3000 (14)	141.0 (14)

Symmetry codes: (i) 
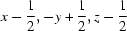
; (ii) 
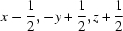
; (iii) 
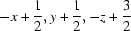
.

**Fig. 1.**
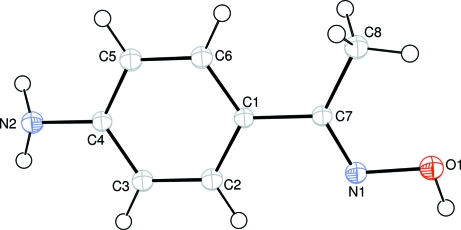
The molecular structure of the title molecule, with the atom-numbering scheme. Displacement ellipsoids are drawn at the 50% probability level.

**Fig. 2.**
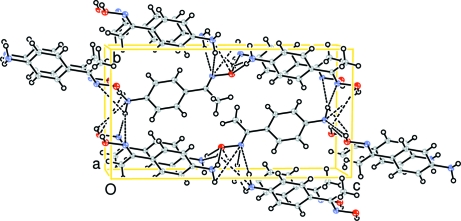
A partial packing diagram. Hydrogen bonds are shown as dashed lines.

## supplementary crystallographic information

### Comment

One of the richest sources of diversity for the medicinal chemist is small heterocyclic rings, which in addition to often exhibiting biological activity, may serve as rigid scaffolds for a further display of functionalities. Oximes are among those, that have been reported to posses a wide range of biological activities including anti-oxidants, anti-inflammatory and as reactivators of organophosphate inhibited acetylcholine esterases (Degorre *et al.*, 1998). The oxime moiety can both donate and accept hydrogen bonds, which makes it a very interesting building block in supramolecular chemistry (Bertolasi *et al.*,  1982). It is also a key intermediate, which undergoes the 1,3-dipolar cycloaddition reaction with mono substituted alkenes to form isoxazolines.Due to importance of these compounds, we decided to synthesize the title compound and report herein its crystal structure.

In the title compound (Fig. 1), the bond lengths (Allen *et al.*, 1987) and angles are within normal ranges . The phenyl ring A (C1-C6) is oriented with respect to the planar (O1/N1/C1/C7/C8) moiety at a dihedral angle of 5.46 (3)°. N2 atom is -0.014 (3) Å away from the phenyl plane.

In the crystal structure, intermolecular O-H···N and N-H···O hydrogen bonds (Table 1) link the molecules to form a supramolecular structure (Fig. 2), in which they may be effective in the stabilization of the structure.

### Experimental

For the preparation of the title compound, a solution of 1-(4-aminophenyl)- ethanone (1.35 g, 10 mmol) in methanol (15 ml) was added to a mixture of hydroxylamine sulfate (1.31 g, 10 mmol) and sodium acetate (2.0 g, 25 mmol). The reaction mass was refluxed for 4-5 h, until the reaction completed. The solvent was evaporated under vacuo and demineralized water (40 ml) was added, cooled to 268-265 K and filtered to obtain crystalline solid (yield; 1.11 g, 75%; m.p. 401-402 K).

### Refinement

H atoms (for OH and NH_2_) were located in difference syntheses and refined isotropically [O-H = 0.92 (2) Å; U_iso_(H) = 0.049 (5) Å^2^ and N-H = 0.916 (18) and 0.929 (19) Å; U_iso_(H) = 0.035 (4) and 0.039 (4) Å^2^]. The remaining H atoms were positioned geometrically, with C-H = 0.95 and 0.98 Å for aromatic and methyl H, respectively, and constrained to ride on their parent atoms with U_iso_(H) = xU_eq_(C), where x = 1.5 for methyl H and x = 1.2 for aromatic H atoms.

### Crystal data

**Table:**

C_8_H_10_N_2_O	*F*(000) = 320
*M**_r_* = 150.18	*D*_x_ = 1.306 Mg m^−^^3^
Monoclinic, *P*2_1_/*n*	Melting point: 401(1) K
Hall symbol: -P 2yn	Mo *K*α radiation, λ = 0.71073 Å
*a* = 4.8641 (2) Å	Cell parameters from 3781 reflections
*b* = 9.2016 (3) Å	θ = 1.0–30.0°
*c* = 17.1447 (7) Å	µ = 0.09 mm^−^^1^
β = 95.535 (2)°	*T* = 100 K
*V* = 763.78 (5) Å^3^	Block, pale yellow
*Z* = 4	0.34 × 0.28 × 0.26 mm

### Data collection

**Table:**

Enraf–Nonius KappaCCD diffractometer	1761 independent reflections
Radiation source: fine-focus sealed tube	1483 reflections with *I* > 2σ(*I*)
horizontally mounted graphite crystal	*R*_int_ = 0.026
Detector resolution: 9 pixels mm^-1^	θ_max_ = 27.5°, θ_min_ = 4.2°
φ and ω scans	*h* = −6→6
Absorption correction: multi-scan (DENZO; Otwinowski & Minor, 1997)	*k* = −11→11
*T*_min_ = 0.972, *T*_max_ = 0.979	*l* = −22→22
6132 measured reflections	

### Refinement

**Table:**

Refinement on *F*^2^	Primary atom site location: structure-invariant direct methods
Least-squares matrix: full	Secondary atom site location: difference Fourier map
*R*[*F*^2^ > 2σ(*F*^2^)] = 0.040	Hydrogen site location: mixed
*wR*(*F*^2^) = 0.108	H atoms treated by a mixture of independent and constrained refinement
*S* = 1.04	*w* = 1/[σ^2^(*F*_o_^2^) + (0.0554*P*)^2^ + 0.2855*P*] where *P* = (*F*_o_^2^ + 2*F*_c_^2^)/3
1761 reflections	(Δ/σ)_max_ < 0.001
113 parameters	Δρ_max_ = 0.24 e Å^−^^3^
0 restraints	Δρ_min_ = −0.28 e Å^−^^3^

### Special details

**Table:**

Geometry. All e.s.d.'s (except the e.s.d. in the dihedral angle between two l.s. planes) are estimated using the full covariance matrix. The cell e.s.d.'s are taken into account individually in the estimation of e.s.d.'s in distances, angles and torsion angles; correlations between e.s.d.'s in cell parameters are only used when they are defined by crystal symmetry. An approximate (isotropic) treatment of cell e.s.d.'s is used for estimating e.s.d.'s involving l.s. planes.
Refinement. Refinement of *F*^2^ against ALL reflections. The weighted *R*-factor *wR* and goodness of fit *S* are based on *F*^2^, conventional *R*-factors *R* are based on *F*, with *F* set to zero for negative *F*^2^. The threshold expression of *F*^2^ > σ(*F*^2^) is used only for calculating *R*-factors(gt) *etc*. and is not relevant to the choice of reflections for refinement. *R*-factors based on *F*^2^ are statistically about twice as large as those based on *F*, and *R*- factors based on ALL data will be even larger.

### Fractional atomic coordinates and isotropic or equivalent isotropic displacement parameters (Å^2^)

**Table:**

	*x*	*y*	*z*	*U*_iso_*/*U*_eq_	
O1	0.42570 (18)	0.21270 (10)	0.47528 (5)	0.0203 (2)	
H1O	0.273 (4)	0.158 (2)	0.4589 (11)	0.049 (5)*	
N1	0.38949 (19)	0.23108 (10)	0.55574 (5)	0.0168 (2)	
N2	0.4407 (2)	0.42572 (11)	0.91465 (6)	0.0186 (2)	
H2N	0.288 (4)	0.3807 (18)	0.9308 (10)	0.035 (4)*	
H2M	0.440 (4)	0.525 (2)	0.9257 (10)	0.039 (4)*	
C1	0.5281 (2)	0.34967 (12)	0.67458 (6)	0.0147 (2)	
C2	0.3462 (2)	0.26638 (12)	0.71490 (7)	0.0179 (3)	
H2	0.2421	0.1914	0.6878	0.021*	
C3	0.3148 (2)	0.29074 (13)	0.79324 (7)	0.0177 (3)	
H3	0.1903	0.2327	0.8192	0.021*	
C4	0.4655 (2)	0.40050 (12)	0.83408 (6)	0.0156 (2)	
C5	0.6512 (2)	0.48187 (13)	0.79535 (7)	0.0187 (3)	
H5	0.7588	0.5550	0.8230	0.022*	
C6	0.6810 (2)	0.45726 (13)	0.71660 (7)	0.0178 (3)	
H6	0.8075	0.5146	0.6910	0.021*	
C7	0.5571 (2)	0.32441 (12)	0.59009 (6)	0.0149 (2)	
C8	0.7713 (2)	0.40782 (13)	0.55104 (7)	0.0195 (3)	
H8A	0.7606	0.3815	0.4954	0.029*	
H8B	0.9553	0.3841	0.5762	0.029*	
H8C	0.7377	0.5123	0.5560	0.029*	

### Atomic displacement parameters (Å^2^)

**Table:**

	*U*^11^	*U*^22^	*U*^33^	*U*^12^	*U*^13^	*U*^23^
O1	0.0222 (5)	0.0272 (5)	0.0121 (4)	−0.0020 (4)	0.0043 (3)	−0.0033 (3)
N1	0.0190 (5)	0.0200 (5)	0.0118 (5)	0.0020 (4)	0.0038 (4)	−0.0010 (4)
N2	0.0230 (5)	0.0190 (5)	0.0141 (5)	0.0005 (4)	0.0032 (4)	−0.0012 (4)
C1	0.0144 (5)	0.0144 (5)	0.0156 (5)	0.0029 (4)	0.0024 (4)	0.0005 (4)
C2	0.0191 (6)	0.0168 (5)	0.0178 (5)	−0.0026 (4)	0.0018 (4)	−0.0015 (4)
C3	0.0172 (5)	0.0182 (6)	0.0181 (6)	−0.0017 (4)	0.0042 (4)	0.0018 (4)
C4	0.0168 (5)	0.0160 (5)	0.0138 (5)	0.0042 (4)	0.0012 (4)	0.0003 (4)
C5	0.0200 (6)	0.0173 (6)	0.0187 (6)	−0.0025 (4)	0.0017 (4)	−0.0036 (4)
C6	0.0179 (5)	0.0176 (5)	0.0185 (6)	−0.0017 (4)	0.0041 (4)	0.0006 (4)
C7	0.0139 (5)	0.0158 (5)	0.0153 (5)	0.0039 (4)	0.0027 (4)	0.0019 (4)
C8	0.0177 (5)	0.0231 (6)	0.0182 (5)	−0.0019 (4)	0.0045 (4)	0.0010 (4)

### Geometric parameters (Å, °)

**Table:**

O1—N1	1.4175 (11)	C3—C4	1.3961 (16)
O1—H1O	0.92 (2)	C3—H3	0.9500
N1—C7	1.2869 (15)	C4—C5	1.3905 (16)
N2—C4	1.4173 (14)	C5—C6	1.3903 (15)
N2—H2N	0.916 (18)	C5—H5	0.9500
N2—H2M	0.929 (19)	C6—H6	0.9500
C1—C6	1.3961 (16)	C7—C8	1.5026 (15)
C1—C2	1.4020 (16)	C8—H8A	0.9800
C1—C7	1.4872 (14)	C8—H8B	0.9800
C2—C3	1.3846 (15)	C8—H8C	0.9800
C2—H2	0.9500		
			
N1—O1—H1O	100.9 (11)	C3—C4—N2	121.15 (10)
C7—N1—O1	113.10 (9)	C6—C5—C4	120.63 (10)
C4—N2—H2N	111.6 (10)	C6—C5—H5	119.7
C4—N2—H2M	111.1 (11)	C4—C5—H5	119.7
H2N—N2—H2M	111.2 (15)	C5—C6—C1	121.09 (10)
C6—C1—C2	117.60 (10)	C5—C6—H6	119.5
C6—C1—C7	121.17 (10)	C1—C6—H6	119.5
C2—C1—C7	121.23 (10)	N1—C7—C1	115.76 (9)
C3—C2—C1	121.59 (11)	N1—C7—C8	124.95 (10)
C3—C2—H2	119.2	C1—C7—C8	119.29 (10)
C1—C2—H2	119.2	C7—C8—H8A	109.5
C2—C3—C4	120.12 (10)	C7—C8—H8B	109.5
C2—C3—H3	119.9	H8A—C8—H8B	109.5
C4—C3—H3	119.9	C7—C8—H8C	109.5
C5—C4—C3	118.94 (10)	H8A—C8—H8C	109.5
C5—C4—N2	119.85 (10)	H8B—C8—H8C	109.5

### Hydrogen-bond geometry (Å, °)

**Table:**

*D*—H···*A*	*D*—H	H···*A*	*D*···*A*	*D*—H···*A*
O1—H1O···N2^i^	0.92 (2)	1.88 (2)	2.7919 (14)	169.8 (18)
N2—H2N···O1^ii^	0.916 (18)	2.165 (18)	3.0790 (13)	175.7 (15)
N2—H2M···N1^iii^	0.929 (19)	2.525 (19)	3.3000 (14)	141.0 (14)

Symmetry codes: (i) *x*−1/2, −*y*+1/2, *z*−1/2; (ii) *x*−1/2, −*y*+1/2, *z*+1/2; (iii) −*x*+1/2, *y*+1/2, −*z*+3/2.
